# Effects of export sophistication on marine pollution: Evidence from Chinese coastal regions

**DOI:** 10.1371/journal.pone.0331881

**Published:** 2026-01-16

**Authors:** Xina Ji, Xingong Ding, Shuping Lin, Xiaoxuan Xie

**Affiliations:** 1 College of International Economics and Trade, Ningbo University of Finance and Economics, Ningbo, Zhejiang, China; 2 College of Economics and Management, Beibu Gulf University, Qinzhou, China; 3 Department of International Trade, Jeonbuk National University, Jeonju, Republic of Korea; Gujarat Institute of Desert Ecology, INDIA

## Abstract

Trade liberalization has accelerated export growth in China’s coastal regions, becoming a major driving force behind local economic development. However, this rapid expansion has also imposed severe environmental pressures on marine ecosystems. As a key indicator for assessing the technological content and quality level of export structures, export sophistication warrants in-depth investigation regarding its potential impact on the marine environment. Drawing on panel data from 11 coastal regions in China between 2011 and 2022, this study employs a two-way fixed-effects model to empirically examine the relationship between export sophistication and marine pollution. The baseline regression results show that higher export sophistication significantly reduces marine pollution. The System GMM dynamic panel analysis further confirms a lagged, long-term pollution-reducing effect. Moreover, environmental regulation, port size, and industrial structure weaken this effect, indicating that the environmental benefits of export sophistication diminish under stricter regulatory conditions, larger port scales, or more advanced industrial structures. The mediation analysis reveals that green technological innovation partially transmits the impact of export sophistication on pollution reduction. Robustness checks, including the exclusion of provinces with extreme pollution levels, confirm the stability of the findings. Heterogeneity analysis further shows that the effects vary across different types of marine pollutants. This study enriches the research perspective on the link between export sophistication and environmental pollution, providing theoretical foundations and policy implications for promoting coordinated development between export structure optimization and marine environmental governance.

## 1. Introduction

Since China’s accession to the WTO in 2001, trade liberalization has steadily advanced, further opening the domestic market and significantly accelerating the growth of import and export activities. As a result, China has gradually evolved into one of the world’s most prominent export-oriented economies [1]. Export trade has become a key driving force behind the country’s economic growth [2,3]. However, this economic achievement has come at a considerable environmental cost to the marine ecosystem. The continuous expansion of exports has markedly accelerated the process of industrialization, with rapid growth observed in manufacturing, transportation, resource extraction, and maritime shipping sectors. This has intensified and broadened industrial production activities, resulting in substantial discharges of waste and pollutants [4,5]. A significant portion of these pollutants ultimately flow into the ocean, posing a severe threat to the health of marine ecosystems [6]. Today, marine pollution has emerged as an urgent environmental challenge, particularly in coastal regions where economic development and societal well-being are heavily dependent on the integrity and stability of marine ecosystems.

From an ecological perspective, marine pollution not only damages the marine ecological environment but also poses severe threats to marine biodiversity and human health. Marine pollution consists of a wide range of contaminants, including potentially toxic metals, hazardous chemical substances, discarded plastics, biomedical waste, and domestic pollutants. These substances are mainly generated on land and are eventually transported into the ocean [7–9]. Once large quantities of land-based pollutants, heavy metals, organic chemicals, and plastic debris enter the ocean, they can lead to eutrophication, red tide outbreaks, and the degradation of ecological habitats, thereby destabilizing the balance of marine ecosystems [10,11]. In addition, secondary pollution caused by port and shipping activities, such as oil contamination, ballast water discharge, and chemical leakage, further exacerbates the deterioration of the coastal environment [12]. These ecological disturbances weaken the resilience of fishery resources and marine ecosystems, posing long-term threats to food security and human well-being in coastal areas. Therefore, a deeper understanding of marine pollution requires the establishment of an integrated analytical framework that links economic activities with marine environmental degradation, underscoring the urgent need to balance trade expansion with marine environmental sustainability.

Against this backdrop, the impact of export product structure on the quality of national economic development has received increasing attention. The export of high-tech-intensive products is widely regarded as conducive to achieving higher-quality economic growth [13]. A key metric in this context is export sophistication, which serves as a core indicator for evaluating the quality of a country’s export structure. This measure is based on the average income level associated with exported products [5]. By assessing the average income linked to a region’s export bundle, export sophistication reflects the technological content and quality level of exported goods. Exporting higher-quality products not only enhances the international competitiveness of a country’s export structure but also enables greater returns in global markets [14]. Therefore, export sophistication is not only an important tool for measuring technological advancement but also a strategic indicator of a country’s position in the global division of labor, playing a critical role in fostering high-quality and sustainable economic development [15]. Existing studies have begun to examine the relationship between export sophistication and the environment, but the focus has primarily been on carbon emissions. For instance, Huang et al. (2023) [16], using data from 31 major economies between 1996 and 2021, investigated the impact of export sophistication in the new energy sector on CO₂ emissions. Their findings indicate that higher export sophistication contributes to emission reduction, with this effect being more pronounced in developed countries. However, the study also identifies a “crowding-out effect” on domestic technological progress, which may partially offset the environmental benefits. Similarly, Yao (2025) [17], employing a two-step system GMM approach based on data from 1995 to 2020, finds an inverted U-shaped relationship between export sophistication and carbon emissions: in the early stages, rising sophistication intensifies emissions, but once a certain threshold is crossed, it facilitates emission reduction, reflecting a structural transition toward cleaner technologies. Nonetheless, there remains a lack of systematic research on the impact of export sophistication on marine pollution. Given that China’s core export regions are predominantly located along the coast, which are particularly vulnerable to marine pollution, whether the upgrading of export structure can help alleviate marine environmental pressure in these regions warrants urgent and in-depth investigation.

Building on this foundation, this study employs a two-way fixed-effects model to systematically analyze the relationship between export sophistication and marine pollution across 11 coastal regions in China from 2011 to 2022. The empirical results indicate that enhancing export sophistication contributes to mitigating marine pollution. Further mechanism analysis reveals that environmental regulations, port scale, and industrial structure exert a negative moderating effect on this relationship, suggesting that the environmental benefits of export structure transformation are largely influenced by institutional policies, port development, and industrial composition. Moreover, the study uncovers heterogeneous effects of export sophistication on different types of pollutant emissions: its mitigating effect is most pronounced for Chemical Oxygen Demand (COD), followed by Ammonia Nitrogen (NHN); the inhibitory effect on Petroleum (PET) is relatively weak, while the impact on Total Phosphorus (TP) is statistically insignificant. This research not only broadens the perspective on the nexus between export sophistication and environmental pollution but also provides valuable insights for governments to design differentiated and targeted environmental governance measures and export structure optimization policies.

This study makes significant contributions on both theoretical and practical fronts. Theoretically, it first expands the scope of research on the trade–environment nexus by introducing export sophistication into the analytical framework of marine environmental studies. This shifts the focus from traditional concerns with carbon emissions or land-based pollution to the domain of marine pollution, thereby enriching the theoretical understanding of the interaction between trade structure upgrading and environmental quality. Second, by incorporating moderating variables such as environmental regulation, port size, and industrial structure, the study systematically reveals the mechanisms through which institutional settings, port infrastructure, and structural conditions influence the relationship between export upgrading and marine pollution. This deepens our understanding of how trade structure optimization can affect environmental performance through institutional, infrastructural, and industrial pathways. Finally, by introducing green technological innovation as a mediating variable, the study empirically tests the mediation effect whereby export sophistication influences marine pollution through the enhancement of green innovation, forming a transmission mechanism of “export sophistication→green innovation→pollution reduction.” Practically, this study provides robust empirical evidence to support marine environmental governance and export structure optimization in coastal regions. The findings indicate that enhancing export sophistication not only contributes to high-quality economic growth but also helps alleviate marine pollution pressures to some extent, offering a feasible path toward coordinated economic and environmental development. Moreover, the moderation analysis shows that the effectiveness of export upgrading in improving environmental outcomes is significantly shaped by the strength of environmental regulation, the level of port development, and the characteristics of industrial structure. This provides important insights for governments in designing region-specific environmental and industrial policies. In addition, the study uncovers heterogeneous effects of export sophistication on different types of pollutants, underscoring the need for differentiated and targeted pollution control strategies based on pollutant characteristics. Overall, the conclusions offer practical implications and policy references for China’s coastal regions and other emerging economies aiming to promote export upgrading while ensuring the protection of marine ecosystems.

The remainder of this study is structured as follows: Section 2 reviews the relevant literature; Section 3 outlines the research methodology; Section 4 presents the empirical findings, including baseline regressions, mechanism analysis, and heterogeneous effects; and Section 5 summarizes the main conclusions and offers targeted policy recommendations based on the results.

## 2. Literature review

### 2.1. Related research on marine pollution

Marine pollution is characterized by multiple input pathways and complex causal mechanisms. Its primary sources include microplastic degradation, poor waste management, and the continuous growth in plastic consumption [18–20] Pourebrahimi and Pirooz, 2023; Onyena et al., 2021; Mihai et al., 2022). Additionally, aquaculture activities [21] and the re-exposure of historical waste in sand dunes [22] have also been identified as significant contributors. Persistent organic pollutants (such as chlorinated paraffins) can enter the ocean via riverine inputs and atmospheric transport, leading to bioaccumulation and trophic transfer [23]. Heavy metal pollution, primarily stemming from industrial, agricultural, and mining activities, poses a severe threat to aquatic ecosystems and human health due to its high toxicity, non-degradability, and tendency to accumulate [24]. Other key risk factors include oil spills caused by shipping accidents and offshore petroleum development, as well as land-based pollution inputs in the absence of effective governance systems ([25–28] Wan et al., 2022; Chenhao and Yupeng, 2021; Wahidul Alam, 2023; Le et al., 2024). In terms of distribution and monitoring, [29] Wang et al. (2023), using data from the Shandong coast between 2014 and 2022, found that the density of floating marine debris in this region exceeds the national average and mainly consists of small- and medium-sized plastic items from human activities, with no significant correlation to precipitation. Mugilarasan et al. (2021) [30], applying the OSPAR standard to the Hooghly Estuary in India, observed increased litter density during the monsoon season, with domestic, tourism, and fishing activities being the primary sources. Mokarram and Pham (2025) [31] employed a combination of NDWI index and K-means clustering to identify oil pollution zones in Iranian waters via remote sensing, achieving high detection accuracy. Moreover, biomarker technologies have proven effective in monitoring the biological impacts of pollution, offering valuable technical support for ecological risk assessment [32]. The ecological and social consequences of marine pollution are increasingly severe. Microplastics accumulate along the food chain in aquatic animals, particularly affecting filter-feeding species such as shellfish, thereby posing risks to human health [33]. Emerging pollutants such as microfibers are highly mobile and bio accumulative [34]. Roman et al. (2021) [35] identified flexible plastics, fishing gear, and balloons as the main items responsible for ingestion-related animal deaths. Heavy metal contamination in marine environments has significantly reduced benthic biodiversity around shipyards [36], while nearshore pollution has led to coastal erosion and geomorphological changes, undermining ecosystem stability [37]. Recent studies have also identified key drivers of marine pollution. Jiang and Li (2021) [38], using panel data, empirically found that political promotion incentives and fiscal decentralization mechanisms weaken local governments’ environmental governance willingness, with a U-shaped relationship observed between pollution levels and officials’ tenure. Ullah et al. (2023) [6], employing time-series data and quantile regression, demonstrated that industrialization and trade liberalization significantly drive industrial wastewater discharge, thereby exacerbating marine pollution. Ji and Ding (2024a) [39], based on multivariate econometric analysis of 11 coastal provinces in China, found that coastal tourism contributes to the reduction of certain pollutants over the long term, although its effect on petroleum-based pollution is insignificant. They also identified a causal relationship between tourism development and pollutant emissions. In terms of policy and institutional responses, Li and Jiang (2023) [40] developed a tripartite game-theoretical model involving the central government, local governments, and marine resource-using enterprises. Their findings suggest that performance evaluation and ecological compensation mechanisms can enhance efficiency of governance. Clayton et al. (2021) [10], in a comparative study of plastic pollution policies across 13 Caribbean countries highlighted that policy tool coordination, public education, and transitional implementation periods are key to effective governance. Zare et al. (2024) [41] emphasized the growing role of artificial intelligence in pollutant detection, oil spill monitoring, and water quality forecasting, identifying it as a crucial technological support for improving marine pollution management capacity.

### 2.2. Related research on export sophistication

Current research on export sophistication primarily focuses on its impact on economic growth. For example, Lazarov and Petreski (2023) [42] employ a dynamic panel system GMM model to analyze panel data from 22 Central and Eastern European countries between 2009 and 2019. Their results show that export sophistication plays a particularly crucial role in promoting economic growth. Furthermore, the study finds that export sophistication not only directly drives economic growth but also enhances export performance through structural transformation. Cardoso et al. (2023) [43], using Brazil as a case study, examine the differential impacts of export and industrial complexity on regional economic growth. Drawing on labor market big data covering 558 micro-regions, they find that while export sophistication is relatively evenly distributed across the country, its effect on economic growth is not significant. This suggests that export sophistication may not adequately capture the knowledge base and growth potential of the Brazilian economy. Abdmoulah (2023) [44], using panel data from 64 countries between 2005 and 2015 and applying a dynamic GMM approach, finds that exports of manufactured goods, high-tech products, and information and communication technology (ICT) products significantly contribute to income growth. Aslanoğlu et al. (2021) [45] measure export sophistication in Middle Eastern and North African countries from 2004 to 2016 and conduct both short- and long-term empirical analyses, comparing OPEC and non-OPEC members. Their findings reveal that export sophistication significantly promotes per capita GDP growth and is one of the key variables explaining fluctuations in economic performance. Chrid et al. (2021) [46] apply a panel cointegration method that accounts for parameter heterogeneity, cross-sectional dependence, and non-stationarity to analyze data from 51 countries spanning 1984–2015. The results indicate a long-run cointegration relationship between export upgrading and economic growth, with a significant positive effect observed in high- and middle-income countries, while no such effect is found in low-income countries. Further causality analysis reveals a long-term bidirectional feedback mechanism between export sophistication and economic growth in high- and middle-income economies. Haini and Pang (2023) [47], from a globalization perspective, explore the moderating role of globalization in the relationship between export sophistication and economic growth. The study finds that in high-income countries, both export sophistication and globalization significantly contribute to growth. However, in developing economies, the marginal effect of export sophistication on growth tends to diminish as the degree of globalization increases. Jarreau and Poncet (2012) [48], using provincial-level data from China for the period 1997–2009, test the theory proposed by Hausmann et al. (2007) [49], which posits that regions specializing in more sophisticated products experience faster economic growth. Their analysis shows that, even after controlling regional development levels, substantial differences in export sophistication exist across provinces and prefecture-level cities, and that higher export sophistication has a significant positive impact on regional economic performance. Lin et al. (2017) [50] focus on Sub-Saharan African countries and find that improvements in export sophistication have a significant income-enhancing effect within countries. Specifically, a 1% increase in export sophistication is associated with a long-term increase of approximately 0.08% in per capita GDP. In addition to economic growth, some studies have also examined the impact of export sophistication on other variables. For instance, Cai and Li (2023) [51] explore the moderating role of export sophistication in the relationship between natural resources and trade diversification. Using a threshold effect model, they find that export sophistication significantly influences the inverted U-shaped relationship between the two: in countries with low export sophistication, natural resources promote trade diversification, while in countries with high sophistication, resource abundance may hinder diversification due to limitations in horizontal specialization. Liu et al. (2022) [52] investigate the relationship between export sophistication and total-factor energy efficiency. Their findings reveal a positive, stage-dependent correlation, indicating that improvements in export sophistication significantly enhance energy efficiency.

It is worth noting that research on the relationship between export sophistication and the environment has been increasing in recent years. Yao (2025) [17] focuses on the link between export sophistication and carbon dioxide emissions, employing a two-step GMM estimation for empirical analysis. The findings reveal an inverted U-shaped relationship between the two. In the early stages, when export sophistication is relatively low, the acceleration of industrialization and heavy reliance on energy-intensive production methods lead to a continuous rise in carbon emissions. However, as the economy advances and more sophisticated production technologies are introduced, emission levels begin to decline, thereby supporting the Environmental Kuznets Curve (EKC) hypothesis. This suggests that although early stages of economic development are often accompanied by environmental degradation, technological progress and improved production efficiency can help mitigate emissions over time. Huang et al. (2023) [16] apply a fixed effects model to examine the impact and underlying mechanisms of export sophistication in the new energy sector on carbon emissions. Their results show that improvements in export sophistication within the new energy industry significantly reduce CO₂ emissions. The underlying logic is that as the capital and technological content of exported new energy products increases, these capital- and technology-intensive goods rely less on fossil fuels compared to traditional products, contributing to the optimization of the energy consumption structure and ultimately to a reduction in carbon emissions. Further mechanism testing reveals that technological advancement acts as a mediating factor in this relationship. However, the study also finds a negative correlation between export sophistication in the new energy sector and the overall level of domestic technological progress. In other words, the rise in export sophistication may exert a crowding-out effect on domestic innovation. This implies that under constrained innovation resources, an export-oriented strategy in the new energy sector may hinder the development of local low-carbon technologies, thereby posing a potential challenge to achieving global carbon reduction goals.

### 2.3. Research gap

This study primarily addresses three research gaps: First, existing literature has largely focused on the impact of export sophistication on carbon emissions, with limited systematic analysis of its relationship with marine pollution. Second, no research to date has explored in depth the specific mechanisms through which export sophistication influences marine pollution. Third, there has been little investigation into the heterogeneous effects of export sophistication on different types of marine pollutants, making it difficult to provide guidance for targeted pollution control. To fill these gaps, this study develops a unified analytical framework to systematically examine the impact of export sophistication on marine pollution, introduces environmental regulation, port size, and industrial structure upgrading as moderating variables to investigate the underlying mechanisms, and conducts a heterogeneity analysis based on pollutant types. The aim is to offer theoretical support and empirical evidence to help coastal regions achieve precise marine pollution management while promoting high-quality foreign trade development.

## 3. Methodology

### 3.1. Econometric model

#### 3.1.1. Benchmark regression model.

To more accurately assess the impact of export sophistication on marine pollution, this study employs a two-way fixed-effects model. This modeling approach is selected because it effectively controls for unobserved regional heterogeneity and time-varying factors, ensuring that the estimated relationship between export sophistication and marine pollution reflects variations within regions rather than overall differences across regions. The model is specified as follows:


$${WWD}_{it}=\alpha_{\text{0}}+\beta_{\text{1}}{EXP}_{it}+\lambda_{\text{1}}{TECH}_{it}+\lambda_{\text{2}}{POP}_{it}+\lambda_{\text{3}}{PGOP}_{it}+\mu_{i}+\nu_{t}+\varepsilon_{it}\;\;\;\;\;\;\;\;$$ (1)

In this model, for coastal province or city *i* in year $t,\;{WWD}_{it}$ denotes the level of marine pollution, ${EXP}_{it}$ represents export sophistication, ${TECH}_{it}$ indicates the level of marine science and technology development, ${POP}_{it}$ is the total population of the coastal province or city, and ${PGOP}_{it}$ reflects the per capita level of marine economic development. $\mu_{i}$ captures region-specific fixed effects, controlling for time-invariant characteristics such as geographic conditions, historical background, and cultural factors. $\nu_{t}$ represents time fixed effects, used to eliminate unobserved factors that vary over time but are common to all regions, such as macroeconomic fluctuations or policy changes. $\varepsilon_{it}$ is the error term, capturing random disturbances not explained by the model.

#### 3.1.2. Moderating effect models.

To further examine the moderating roles of environmental regulation, port size, and industrial structure, this study constructs the following moderating effect models:


$${WWD}_{it}=\alpha_{\text{0}}+\beta_{\text{1}}{EXP}_{it}+\gamma_{\text{1}}{ER}_{it}+\delta_{\text{1}}{EXP}_{it}\times {ER}_{it}+\lambda_{\text{1}}{TECH}_{it}+\lambda_{\text{2}}{POP}_{it}+\lambda_{\text{3}}{PGOP}_{it}+\mu_{i}+\nu_{t}+\varepsilon_{it}$$ (2)

$${WWD}_{it}=\alpha_{\text{0}}+\beta_{\text{1}}{EXP}_{it}+\gamma_{\text{1}}{PORT}_{it}+\delta_{\text{1}}{EXP}_{it}\times {PORT}_{it}+\lambda_{\text{1}}{TECH}_{it}+\lambda_{\text{2}}{POP}_{it}+\lambda_{\text{3}}{PGOP}_{it}+\mu_{i}+\nu_{t}+\varepsilon_{it}\;$$ (3)

$${WWD}_{it}=\alpha_{\text{0}}+\beta_{\text{1}}{EXP}_{it}+\gamma_{\text{1}}{IND}_{it}+\delta_{\text{1}}{EXP}_{it}\times {IND}_{it}+\lambda_{\text{1}}{TECH}_{it}+\lambda_{\text{2}}{POP}_{it}+\lambda_{\text{3}}{PGOP}_{it}+\mu_{i}+\nu_{t}+\varepsilon_{it}$$ (4)

Where, ${ER}_{it}$ denotes the intensity of environmental regulation, and ${EXP}_{it}\times {ER}_{it}$ captures the interaction effect between export sophistication and environmental regulation. ${PORT}_{it}$ represents port size, and ${EXP}_{it}\times {PORT}_{it}$ reflects the interaction between export sophistication and port size. ${IND}_{it}$ indicates industrial structure, and ${EXP}_{it}\times {IND}_{it}$ represents the interaction between export sophistication and industrial structure. All other variables are defined as in Equation (1).

### 3.2. Data and variable description

This study follows the principles of theoretical relevance and data availability in the selection of variables. The dependent variable reflects the level of marine pollution, while the core explanatory variable measures the degree of trade structure upgrading. Moderating variables are used to capture the potential influence of institutional and structural conditions on this relationship, and the mediating variable represents the level of green technological innovation. Control variables are included to account for the confounding effects of other factors such as economic development, population dynamics, and technological progress. All variables are selected with reference to existing literature and based on the availability of reliable data.

#### 3.2.1. Dependent variable.

This study takes marine pollution as the dependent variable and measures it using the total volume of wastewater discharged directly into the sea from pollution sources in China’s coastal regions. The discharge of wastewater into marine systems is an inevitable stage in wastewater treatment, as most terrestrial wastewater ultimately flows into the ocean, making land-based pollution one of the primary contributors to marine pollution [53,54]. Monitoring the volume of wastewater directly discharged into marine outfalls provides a more intuitive and efficient means of assessing the actual state of marine pollution [39]. The relevant data are obtained from the Bulletin of Marine Ecology and Environment Status of China.

#### 3.2.2. Independent variable.

The independent variable in this study is export sophistication. Export sophistication comprehensively reflects the technological content and production efficiency of export products, serving as an effective indicator of a country or region’s trade structure upgrading and the quality of its trade development [49,55,56]. Generally, a higher level of export sophistication indicates that export products have greater added value and, consequently, stronger competitiveness in international markets [57,58]. The advancement of export sophistication not only reflects an expansion in export size but also signifies a dynamic upgrading process in which the technological level of products evolves from basic to advanced stages [59].

Building on relevant literature [58,60,61] (Fatum et al., 2018; Liang and Tan, 2024b; Liu et al., 2023), the export sophistication of China’s coastal regions is calculated in two steps. First, the technological sophistication of each export product category is measured using the methodology proposed by Hausmann et al. (2007) [49]:


$${PEXP}_{kt}=\sum_{i}\frac{\frac{x_{ikt}}{X_{it}}}{\sum_{i}\frac{x_{ikt}}{X_{it}}}Y_{it}\;\;\;\;\;\;$$ (5)

In this equation, ${PEXP}_{kt}$ represents the export sophistication of HS 6-digit product k in year t. $x_{ikt}$ denotes the export value of product k from province or city i in year t, and $X_{it}$ is the total export value of province or city i in year t. $Y_{it}$ refers to the per capita GDP of province or city i in year t. $\frac{x_{ikt}}{X_{it}}$ indicates the share of product k in the total exports of province or city i in year t. $\sum_{i}\frac{x_{ikt}}{X_{it}}$ represents the share of product k in the total exports of all coastal provinces and cities in year t. $\sum_{i}\frac{\frac{x_{ikt}}{X_{it}}}{\sum_{i}\frac{x_{ikt}}{X_{it}}}$ reflects the revealed comparative advantage of region i in exporting product k in year t.

Next, using the share of each product in total exports as weights, a weighted average of product-level export sophistication is calculated to derive the overall export sophistication level for each province or city:


$${EXP}_{it}=\sum_{k}\frac{x_{ikt}}{X_{it}}{PEXP}_{kt}\;\;$$ (6)

Where ${EXP}_{it}$ denotes the export sophistication of province or city i in year t.

#### 3.2.3. Moderating variables.

To account for the mechanisms through which export sophistication affects marine pollution, this study introduces three moderating variables: environmental regulation, port size, and industrial structure.

First, environmental regulation refers to government supervision and management of local production and business activities through measures such as administrative orders, emission permits, pollution taxes, and administrative penalties, with the core objective of balancing economic development and environmental protection [62]. Following [63] Zeng et al., (2019), this study adopts the total investment in industrial pollution control as a proxy variable for environmental regulation. As investment in pollution control continues to increase, the pollution treatment equipment and technologies introduced by the government become more advanced, thereby improving overall treatment efficiency [64]. Consequently, stronger environmental regulation can effectively enhance the ability to control pollutant emissions, reduce the discharge of land-based pollutants into the ocean, and improve ecological environmental quality in coastal regions [65]. The relevant data are obtained from the China Statistical Yearbook.

Second, as vital economic and transportation hubs in coastal regions, ports not only provide logistics and service support to cargo owners, shipping companies, and other stakeholders but also play a key role as engines driving regional economic development [66,67]. Among various indicators, port cargo throughput is an essential measure of port activity, referring to the total volume of goods entering and leaving a port area via waterways and undergoing loading and unloading operations within a given period. This metric effectively reflects a port’s operational status and overall scale [68]. Moreover, the flow direction, cargo composition, and volume characteristics of port throughput reveal the port’s position and influence within regional and even global shipping networks [69]. Therefore, following [70] Ding et al. (2025), this study uses the cargo throughput of coastal provinces and municipalities as a representative indicator of port scale, with data sourced from the China Port Yearbook.

Finally, industrial structure upgrading reflects the dynamic process by which production factors shift from industries with low value-added, low efficiency, and high resource consumption to those with high-value-added, high efficiency, and low-resource-consumption, serving as an important driving force for promoting high-quality economic development [71]. Building on this, industrial structure upgrading can be further understood as the evolution and optimization of the industrial structure toward higher efficiency and more optimal resource allocation [72]. This study uses the proportion of value added in secondary industry to GDP as a proxy variable for industrial structure. The relevant data are also sourced from the China Statistical Yearbook.

#### 3.2.4. Mediating variable.

To examine the mechanism through which export sophistication affects marine pollution, this study draws on the approaches of Tiwari and Si Mohammed (2024) and Xiang and Geng (2024) [73,74], and introduces green patents as a mediating variable to measure the level of green technological innovation (GRE) in coastal regions. GRE comprehensively reflect regional capabilities in energy conservation, clean production, and ecological protection, and thus serve as an important indicator of green innovation activities. The relevant data are obtained from the China Statistical Yearbook. By incorporating this variable, we depict the transmission pathway whereby export sophistication promotes green technological innovation and subsequently contributes to pollution reduction, thereby verifying the mediating mechanism of “export sophistication→green technological innovation→pollution mitigation.”

#### 3.2.5. Control variables.

To avoid estimation bias caused by omitted variables, this study includes several control variables to comprehensively assess the potential factors influencing marine pollution. Marine scientific and technological innovation plays a vital role in shifting the development of marine resources from extensive to intensive modes, promoting sustainable growth of the marine economy, and enhancing the efficiency of marine environmental governance, thereby improving ecological quality [75]. Accordingly, this study uses the number of marine R&D institutions as a proxy for the level of marine scientific and technological innovation. Population size is another important determinant of environmental pressure and is typically measured by the total population of each province each year [76]. In line with this, the annual total population of each province or city is used to capture population scale. Gross Ocean Product (GOP) refers to the final output generated by resident units in coastal areas through ocean-related economic activities over a given period, calculated at market prices. It is equal to the sum of value added from the primary, secondary, and tertiary marine industries and serves as a comprehensive indicator of a region’s level of marine economic development and service capacity [77,78]. Following the approach of [79] Shao et al. (2021), this study adopts real per capita GOP as the indicator for regional marine economic development. Data are sourced from the China Marine Statistical Yearbook. Table 1 presents the variable sources and descriptions, and Table 2 reports the descriptive statistics.

**Table 1:** Variable source and description.

Variables	Symbols	Specification	Data source
Wastewater discharge	WWD	Wastewater discharge from direct marine pollution sources	Bulletin of Marine Ecology and Environment Status of China
Chemical oxygen demand	COD	Chemical oxygen demand discharge from direct marine pollution sources	Bulletin of Marine Ecology and Environment Status of China
Ammonia nitrogen	NHN	Ammonia nitrogen discharge from direct marine pollution sources	Bulletin of Marine Ecology and Environment Status of China
Petroleum	PET	Petroleum discharge from direct marine pollution sources	Bulletin of Marine Ecology and Environment Status of China
Total phosphorus	TP	Total phosphorus discharge from direct marine pollution sources	Bulletin of Marine Ecology and Environment Status of China
Export sophistication	EXP	Export sophistication index	Author’s calculation
Environmental regulation	ER	Logarithm of completed investment in industrial pollution treatment	China Statistical Yearbook
Green technological innovation	GRE	Green utility model patents/total green patents	China Statistical Yearbook
Port size	PORT	Logarithm of port cargo throughput	China Marine Statistical Yearbook
Industrial structure	IND	Secondary industry value added/ GDP	China Statistical Yearbook
Technological innovation	TECH	Logarithm of the number of marine R&D institutions	China Marine Statistical Yearbook
Population size	POP	Logarithm of total population	China Statistical Yearbook
Per capita gross ocean product	PGOP	GOP/total population	China Marine Statistical Yearbook

**Table 2:** Descriptive statistics.

Variables	Obs.	Mean	Std. dev.	Min	Max
WWD	132	0.612	0.677	0.019	3.254
COD	132	1.649	1.964	0.057	8.700
NHN	132	0.100	0.129	0.001	0.800
PET	132	0.008	0.011	0.000	0.069
TP	132	0.020	0.019	0.001	0.087
EXP	132	9.117	0.207	8.679	9.552
ER	132	12.061	1.251	6.165	14.164
PORT	132	11.022	0.750	9.297	12.150
IND	132	0.402	0.083	0.190	0.528
GRE	132	0.523	0.106	0.205	0.713
TECH	132	2.541	0.583	1.099	3.689
POP	132	8.392	0.768	6.791	9.448
PGOP	132	1.431	1.043	0.132	4.194

## 4. Results

### 4.1. Baseline regression results

Table 3 presents the baseline regression results of the impact of export sophistication on marine pollution in coastal regions. Regression models (1) through (3) sequentially include control variables such as the level of marine science and technology development, population size, and per capita gross ocean product to assess the robustness of the findings. The core explanatory variable, export sophistication, exhibits a consistently significant negative effect across all three models, with coefficients of −1.684, −1.672, and −1.694, all statistically significant at the 5 percent level. These results indicate that an increase in the technological sophistication of export products significantly contributes to the reduction of marine pollution, suggesting that a shift toward an export structure characterized by higher value-added and technologically advanced products is a critical pathway for improving the marine ecological environment. The underlying reasons are as follows. On the one hand, an increase in export sophistication can drive the optimization and upgrading of regional production structures, with traditional heavy industries gradually being replaced by high-tech industries, thereby accelerating the widespread adoption of environmentally friendly and innovative technologies [17]. This transformation helps reduce the discharge of industrial wastewater and other land-based pollutants at the source, contributing positively to the restoration and protection of marine ecosystems. On the other hand, higher export sophistication fosters the development of clean energy and green industries [16]. As the technological content and refinement level of export products increase, local governments are more inclined to allocate resources toward infrastructure improvement and upgrading, thereby enhancing energy efficiency, reducing reliance on energy-intensive production methods, and mitigating the risk of pollutants entering the marine environment.

**Table 3:** Baseline regression results.

Variables	(1)	(2)	(3)
EXP	−1.684^**^ (0.650)	−1.672^**^ (0.634)	−1.694^**^ (0.602)
TECH	0.273^***^ (0.073)	0.202^***^ (0.092)	0.206^**^ (0.085)
POP		1.935 (1.093)	1.956 (1.098)
PGOP			−0.021 (0.091)
Constant	14.480^**^ (5.569)	−1.585 (11.070)	−1.555 (10.848)
N	132	132	132

Note: The values in parentheses are clustered robust standard errors. ^*^, ^**^, and ^***^denote significance at the 10%, 5%, and 1% levels, respectively.

The level of marine science and technology development exhibits a significant positive effect across all models, with results statistically significant at the 1 percent level. This suggests that current investments in scientific and technological advancement have not yet been fully translated into environmental governance capacity in the short term. Regarding population size, models (2) and (3) both show a significant positive effect, passing the 5 percent significance threshold, indicating that population growth intensifies land-based pollutant emissions and thereby increases pressure on the marine environment in coastal areas. The coefficient for per capita gross ocean product is negative but not statistically significant, suggesting that the level of per capita marine economic development does not have a notable impact on marine pollution.

### 4.2. Dynamic panel estimation results

To address the potential endogeneity between EXP and WWD and enhance the reliability of causal identification, this study further employs the System GMM dynamic panel model for estimation. Table 4 presents the System GMM estimation results. Based on the Arellano-Bond test, the optimal lag order of the pollution variable is determined to be two. Accordingly, the first lag of marine pollution (L1.WWD) is included as a dynamic term to capture the persistence of pollution. In addition, the first (L1.EXP) and second lags (L2.EXP) of export sophistication are treated as endogenous variables to mitigate potential simultaneity bias. The estimation results indicate that the coefficient of L1.WWD is 0.683 and statistically significant at the 10% level, confirming the persistence of marine pollution. The coefficients of current-period EXP and L1.EXP are statistically insignificant, while the coefficient of L2.EXP is −0.263 and significant at the 1% level, suggesting that improvements in export sophistication lead to a significant reduction in marine pollution with a two-period lag. This finding supports the hypothesis that export sophistication has a long-term effect on improving marine environmental quality. Model diagnostic tests further confirm the validity of the specification. The p-value of the Arellano-Bond first-order autocorrelation test (AR(1)) is 0.055, indicating appropriate negative first-order serial correlation in the differenced residuals. The second-order autocorrelation test (AR(2)) yields a p-value of 0.617, suggesting the absence of second-order autocorrelation. The Hansen test returns a p-value of 0.999, indicating the overall validity of the instruments and no evidence of overidentification. These results confirm the reliability of the System GMM estimation.

**Table 4:** System GMM dynamic panel results.

Variables	GMM-system
L1. WWD	0.683^*^ (0.412)
EXP	0.467 (0.564)
L1. EXP	0.107 (0.259)
L2. EXP	−0.263^***^ (0.028)
TECH	0.041 (0.113)
POP	−0.104*** (0.026)
PGOP	−0.227 (0.175)
Constant	−1.555 (3.205)
N	110
Arellano-Bond AR (1)	0.055
Arellano-Bond AR (2)	0.617
Hansen test	0.999

Note: The values enclosed in parentheses represent standard errors. L1 and L2 represent the first-order and second-order lag terms, respectively. AR (1), AR (2), and Hansen show P values. ^*^, ^**^, and ^***^denote significance at the 10%, 5%, and 1% levels, respectively.

### 4.3. Moderating effects results

To further investigate the mechanisms through which export sophistication affects marine pollution, this study introduces environmental regulation, port size, and industrial structure as moderating variables, and constructs interaction terms for empirical testing.

The results of Model (2) show that the interaction term between export sophistication and environmental regulation has a coefficient of 0.215, which is statistically significant at the 5% level. This indicates that environmental regulation exerts a negative moderating effect on the relationship between export sophistication and marine pollution. In other words, in regions with stronger environmental regulation, the pollution-reducing effect of export sophistication is somewhat weakened. Under strict regulatory conditions, coastal regions commonly adopt pollution treatment technologies [80], which diminishes the incremental environmental improvements brought by technological sophistication. In Model (4), the interaction term between export sophistication and port scale (EXP × PORT) has a coefficient of 0.370, which is statistically significant at the 1% level. This indicates that an expansion in port scale weakens the positive effect of export sophistication on marine environmental improvement. The expansion of ports leads to increased transportation activities, resulting in higher pollution emissions and a pollution agglomeration effect [81], which partially offsets the emission reduction benefits of high-technology-intensive exports. The results of Model (6) show that the interaction term between export sophistication and industrial structure (EXP × IND) has a coefficient of 3.509, which is statistically significant at the 1% level. This indicates that industrial structure upgrading also weakens the positive impact of technological progress on marine environmental improvement. Industrial structure upgrading represents the shift of production factors from low value-added, low-efficiency, and high-consumption sectors toward high-value-added, high-efficiency, and low-consumption sectors, accompanied by a gradual reduction in high-pollution and high-energy-consuming industries, thereby contributing to emission reduction [71,82]. However, this also implies that as industrial structure optimization progresses, the additional environmental improvements attainable through export sophistication become increasingly limited.

Overall, these findings suggest that the moderating effects of environmental regulation, port scale, and industrial structure all weaken the negative relationship between export sophistication and marine pollution, indicating that in stricter regulatory, larger-scale port, or more advanced industrial contexts, the marginal environmental benefits of export sophistication are diminished (Table 5).

**Table 5:** Moderating effects results.

Variables	(1)	(2)	(3)	(4)	(5)	(6)
EXP	−1.616^**^ (0.649)	−3.046^***^ (0.741)	−1.681^**^ (0.593)	−4.577^***^ (0.725)	−1.809^*^ (0.843)	−1.826^**^ (0.750)
ER	0.009 (0.015)	−1.977^***^ (0.547)				
EXP×ER		0.215^***^ (0.060)				
PORT			0.155 (0.165)	−3.078^***^ (0.657)		
EXP×PORT				0.370^***^ (0.070)		
IND					1.166 (3.017)	−31.710^***^ (8.631)
EXP×IND						3.509^***^ (0.813)
TECH	0.206^**^ (0.085)	0.214^**^ (0.068)	0.186^*^ (0.088)	0.200^**^ (0.065)	0.213^*^ (0.096)	0.258^**^ (0.083)
POP	1.933 (1.080)	1.612^*^ (0.869)	1.649 (1.183)	0.908 (0.868)	1.750 (1.364)	1.551 (1.175)
PGOP	−0.022 (0.093)	0.036 (0.072)	0.001 (0.075)	0.067 (0.063)	−0.024 (0.084)	0.034 (0.048)
Constant	−2.161 (11.318)	14.250 (10.261)	−0.750 (11.338)	30.620^***^ (6.824)	0.606 (14.926)	3.253 (13.267)
N	132	132	132	132	132	132

Note: The values in parentheses are clustered robust standard errors. ^*^, ^**^, and ^***^denote significance at the 10%, 5%, and 1% levels, respectively.

### 4.4. Mediation effect results

Table 6 presents the results of the mediation effect analysis, examining how export sophistication influences marine pollution through green technological innovation (GRE).

**Table 6:** Mediation effect results.

Variables	WWD	GRE	WWD
EXP	−1.694^**^ (0.602)	0.738^**^ (0.255)	−1.227^*^ (0.672)
GRE			−0.634^*^ (0.290)
TECH	0.206^**^ (0.085)	0.011 (0.040)	0.213^**^ (0.073)
POP	1.956 (1.098)	−0.983 (0.659)	1.332 (0.739)
PGOP	−0.021 (0.091)	−0.024 (0.030)	−0.036 (0.083)
Constant	−1.555 (10.848)	2.249 (6.388)	−0.130 (7.907)
N	132	132	132

Note: The values in parentheses are clustered robust standard errors. ^*^, ^**^, and ^***^denote significance at the 10%, 5%, and 1% levels, respectively.

Model (1) tests the total effect and shows that the regression coefficient of export sophistication on marine pollution is −1.694, which is statistically significant at the 5% level. This indicates that an increase in export sophistication significantly reduces marine pollution. Model (2) introduces the mediating variable, green technological innovation, as the dependent variable. The results show that the coefficient of export sophistication is 0.738 and significant at the 5% level, suggesting that higher export sophistication significantly promotes green innovation. Model (3) includes both export sophistication and green technological innovation. The coefficient of green innovation on marine pollution is −0.634 and statistically significant at the 10% level, indicating that green innovation contributes to a reduction in marine pollution. Meanwhile, the coefficient of export sophistication remains significant at −1.227, although the absolute value is smaller than in Model (1). This implies that green technological innovation plays a partial mediating role in the relationship between export sophistication and marine pollution. In other words, export sophistication reduces pollution both directly and indirectly by enhancing green innovation, thereby confirming the transmission mechanism of “export sophistication→green technological innovation→pollution reduction” and validating the partial mediation effect of green innovation.

### 4.4. Robustness test results

Table 7 reports the results of the robustness test. To verify the reliability of the baseline regression results, this study excludes Zhejiang Province, which has the highest level of marine pollution, and re-estimates the two-way fixed effects model using data from the remaining 10 coastal provinces. The results show that, after sequentially adding control variables, the impact of export sophistication on marine pollution remains consistently negative and statistically significant. This indicates that the regression results of this study are highly robust.

**Table 7:** Robustness test results.

Variables	(1)	(2)	(3)
EXP	−1.762^**^ (0.586)	−1.745^**^ (0.580)	−1.750^***^ (0.527)
TECH	0.286^***^ (0.072)	0.259^**^ (0.087)	0.259^**^ (0.080)
POP		0.669 (0.593)	0.676 (0.611)
PGOP			−0.005 (0.095)
Constant	15.053^**^ (4.960)	9.401 (7.809)	9.389 (7.860)
N	120	120	120

Note: The values in parentheses are clustered robust standard errors. ^*^, ^**^, and ^***^ indicate significance at the 10%, 5%, and 1% levels, respectively.

### 4.5. Heterogeneity analysis results

Table 8 presents the heterogeneous effects of export sophistication on different types of pollutant emissions. The results show that the regression coefficient of export sophistication on COD is −5.158 and statistically significant at the 1% level, indicating that exports of high-technology-intensive products can significantly reduce organic pollutant emissions. As a key indicator for assessing water quality, chemical oxygen demand (COD) quantifies the amount of organic matter in water. Excessive COD concentrations can exacerbate hypoxic events in shallow waters [83] and directly reflect the extent of industrial activity’s impact on surface water pollution [84]. High-technology exports are typically accompanied by advanced production processes and stricter environmental standards, which reduce industrial wastewater discharges at the source, making the mitigation effect on COD particularly pronounced.

**Table 8:** Heterogeneity of pollutant discharge types.

Variables	COD	NHN	PET	TP
EXP	−5.158^***^ (1.809)	−0.760^***^ (0.280)	−0.044^*^ (0.023)	−0.026 (0.032)
TECH	1.296^***^ (0.176)	0.182^***^ (0.027)	0.009^***^ (0.002)	0.018^***^ (0.003)
POP	−6.135^***^ (2.150)	−1.662^***^ (0.332)	−0.148^***^ (0.027)	−0.215^***^ (0.038)
PGOP	−0.215 (0.188)	0.014 (0.029)	−0.002 (0.002)	0.001 (0.003)
Constant	95.23^***^ (23.624)	20.220^***^ (3.651)	1.602^***^ (0.294)	2.007^***^ (0.413)
N	132	132	132	132
F	135.78	13.56	26.30	21.99
$R^{\text{2}}$	0.382	0.427	0.260	0.538

Note: The values enclosed in parentheses represent standard errors. ^*^, ^**^, and ^***^denote significance at the 10%, 5%, and 1% levels, respectively.

The regression coefficient for NHN is −0.760 and statistically significant at the 1% level, indicating that higher export sophistication helps reduce ammonia nitrogen emissions. Ammonia nitrogen pollution mainly stems from inappropriate fertilizer use as well as uncontrolled discharges of industrial and domestic wastewater [85]. Therefore, while improvements in export sophistication can partially reduce ammonia nitrogen emissions from industrial wastewater, the relatively large contribution of non-industrial sources such as agricultural runoff and domestic sewage makes its mitigation effect weaker compared with that on COD.

For PET, the regression coefficient of export sophistication is −0.044 and significant only at the 10% level, suggesting a relatively limited inhibitory effect on petroleum-based pollutant emissions. Petroleum pollution mainly originates from industrial production, oil spill incidents, and petroleum refining activities [86,87]. Although optimizing the export structure can help reduce fossil fuel consumption at the source and curb part of the emissions, unavoidable pollutant releases during transportation weaken the overall reduction effect, resulting in suboptimal mitigation outcomes.

For TP, the regression coefficient is −0.026 and not statistically significant, indicating that export structure optimization has no notable effect on reducing total phosphorus emissions. Besides effluent discharges from wastewater treatment plants, agricultural activities are among the main contributors to total phosphorus loads [88]. Given the weak connection between these pollution sources and export sophistication, relying solely on improving export sophistication is insufficient for effectively controlling total phosphorus emissions.

## 5. Conclusion and policy implications

Marine pollution has long been a major global environmental concern, and the relationship between international trade and marine pollution has increasingly become a prominent topic of academic research. Using panel data from 11 coastal provinces and municipalities in China covering the period from 2011 to 2022, this study systematically investigates the mechanisms through which export sophistication affects marine pollution and further analyzes its heterogeneous effects on different types of pollutants.

The findings indicate that enhancing export sophistication significantly reduces marine pollutant emissions, supporting the notion that optimizing the export structure plays a positive role in improving marine environmental quality. Specifically, higher technological sophistication drives the upgrading of regional industrial structures, with high-tech industries gradually replacing traditional high-pollution sectors, thereby reducing land-based pollutant discharges at the source. At the same time, technological progress fosters the development of clean energy and green industries, improve energy efficiency, and reduces reliance on high-pollution energy sources, ultimately lowering the risk of pollutants entering the marine environment. To further uncover the underlying mechanisms, this study introduces environmental regulation, port scale, and industrial structure as moderating variables. The results show that in regions with stricter environmental regulations, pollution control technologies are already widely implemented, diminishing the marginal emission-reduction benefits of improved technological sophistication. Similarly, larger port scales increase transportation activities and pollution agglomeration effects, partially offsetting the environmental benefits of higher export sophistication. Industrial structure upgrading also contributes to emission reductions, but as high-pollution industries have already declined, the additional environmental improvements attributable to further sophistication become weaker. Moreover, the effects of export sophistication vary significantly across pollutant types. For COD, high-tech exports effectively reduce organic pollutant emissions due to advanced production processes and stringent environmental standards that minimize industrial wastewater discharge at the source. For NHN, sophistication improvements also help reduce emissions, but their impact is relatively limited given the substantial contributions of agricultural runoff and domestic sewage. For PET, optimizing the export structure can reduce fossil fuel consumption to some extent, but unavoidable emissions during transportation weaken the overall mitigation effect. For TP, pollution primarily originates from agricultural activities and domestic wastewater, which are only weakly linked to the export structure, making improvements in technological sophistication insufficient to effectively control such emissions.

Based on the above findings, this study proposes the following policy recommendations.

First, given the significant effect of export sophistication in mitigating marine pollution, governments should actively promote the transformation of export structures toward high-tech and high-value-added industries. Through fiscal subsidies, tax incentives, and trade policy optimization, authorities can encourage the development of clean production and green exports in coastal regions, thereby reducing land-based pollutant discharges at the source and improving marine environmental quality. Second, in advancing the coordinated development of optimized export structures and marine environmental protection, the alignment between environmental regulations and port development policies should be strengthened. On one hand, environmental regulations should adopt differentiated measures tailored to the industrial characteristics of each coastal region, combining policy guidance with technological support to enhance flexibility and adaptability, thereby avoiding diminishing marginal emission-reduction benefits of technological progress caused by excessive regulatory intensity. On the other hand, efforts should be made to accelerate the construction of green ports, optimize port layout and transportation organization, and upgrade environmental protection facilities to alleviate the pollution agglomeration effects arising from port expansion and increased transportation activities. Finally, considering the heterogeneous effects of export sophistication on different types of pollutants, differentiated pollution control strategies should be developed. For organic pollutants such as COD, stricter supervision and wider adoption of industrial wastewater treatment technologies are needed to reduce emissions at the source. For NHN, while upgrading the export structure, greater efforts should be made to manage agricultural non-point source pollution, promote the use of high-efficiency and low-pollution fertilizers, and continuously improve sewage treatment systems. For petroleum-based pollutants, green transformation of ports and transportation activities should be promoted by adopting clean fuels and new-energy vessels, strengthening anti-leakage measures, and enhancing emergency response mechanisms to mitigate pollution risks from shipping activities. For TP, priority should be given to controlling agricultural emissions, promoting pollution-prevention technologies for non-point sources, and improving the phosphorus removal efficiency of wastewater treatment plants to compensate for the limited impact of export sophistication on this type of pollution.

This study has certain limitations, which suggest potential directions for future research. First, due to constraints in data availability, the study period only covers the years from 2011 to 2022. Although this period provides a meaningful reflection of the relationship between export structure upgrading and marine pollution in China’s coastal regions, the temporal scope is relatively limited. As statistical data continue to improve and become more complete in the future, access to longer time series or higher-quality observational data would help enhance the robustness of the findings and provide a more solid empirical foundation for subsequent research. Second, in terms of mechanism analysis, this study focuses primarily on the moderating effects of environmental regulation, port size, and industrial structure, as well as the mediating role of green technological innovation. However, it does not fully account for other potentially important factors such as energy structure and financial development. Future research may extend the analysis by incorporating a wider range of institutional and structural dimensions to construct a more systematic and multi-level explanatory framework. Finally, as the study is based on data from China, the external applicability of the conclusions remains to be verified. Future studies could conduct cross-national comparative analyses in countries along the Belt and Road Initiative or in other emerging economies, to examine whether the effects of export structure upgrading hold under different ecological and institutional contexts. Such comparative research would further enrich the international evidence base and provide valuable policy implications for aligning export upgrading with marine environmental protection.

## Supporting information

S1 Data Data. (XLSX)
